# Microfluidic-based biomimetic mitochondrial nanocomposite for targeted immunotherapy of rheumatoid arthritis via mitochondrial transplantation

**DOI:** 10.1016/j.mtbio.2026.103371

**Published:** 2026-06-19

**Authors:** Nengjie Yang, Chen Dong, Rui Zhao, Mei Yang, Shiwen Ni, Yi Jin, Qingshui Wen, Cong Xu, Zhifeng Gu, Yujuan Zhu, Chi Sun

**Affiliations:** a Department of Rheumatology, Research Center of Clinical Medicine, Research Center of Immunology, Affiliated Hospital of Nantong University, Medical School of Nantong University, Nantong, 226001, China; b Department of Biomedical Engineering, Columbia University, New York, NY, 10027, USA; c Department of Geriatrics, Research Center of Clinical Medicine, Research Center of Immunology, Affiliated Hospital of Nantong University, Medical School of Nantong University, Nantong, 226001, China

**Keywords:** Biomimetic mitochondrial nanocomposite, Microfluidic chip, Mitochondrial transplantation, Dendritic polylysine, Rheumatoid arthritis

## Abstract

Rheumatoid arthritis (RA) is a common chronic autoimmune disease and has recently been reported to be closely related to mitochondrial dysfunction. Mitochondrial dysfunction can promote the occurrence and development of rheumatoid arthritis (RA) through increased cellular ROS production, activation of immune cells and production of autoantibodies. Although the mechanism of mitochondrial dysfunction remains uncertain, it offers potential therapeutic strategies for rheumatoid arthritis. Mitochondrial transplantation is an emerging treatment method, aiming to restore the normal function of tissues by replacing abnormal mitochondria in tissues or cells. Here, we propose a novel biomimetic mitochondrial nanocomposite (Mito@G3K) based on microfluidic chips, which can be used for intravenous RA targeted immunotherapy. Based on microfluidic chips, Mito@G3K were efficiently generated by taking advantage of the charge capture effect between mitochondria and cationic peptide dendritic macromolecules. Due to the naturally derived peptide components, the synthetic Mito@G3K have very high biological activity. Even more attractive is that by customizing Mito@G3K with a higher surface charge density, they can achieve rapid targeting ability, high accumulation volume and long-lasting effect in inflamed joints. In vitro experiments have shown that it can effectively inhibit the inflammation caused by pro-inflammatory macrophages. In addition, Mito@G3K have shown good therapeutic effects in the CIA mouse model and can effectively alleviate inflammation in the joint area. Mouse synovial transcriptome sequencing shows that the therapeutic effect may be achieved by improving mitochondrial metabolism.

## Graphical abstract

**Figure:**
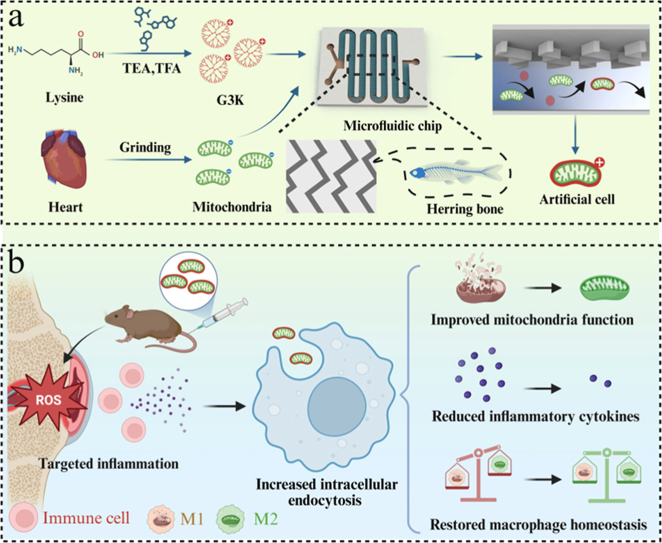

## 1 Introduction

Rheumatoid arthritis (RA) is a prevalent autoimmune disease that predominantly affects middle-aged and older women [1,2]. Chronic joint inflammation in RA can lead to joint damage and disability [3,4].The pathogenesis of RA is complex, with synovial hyperplasia and angiogenesis being key features [5,6]. Although the exact cause remains unclear, a combination of immune cells, cytokines, genetic predispositions, and environmental factors are believed to contribute to its development [7,8]. Current treatments for RA include synthetic disease-modifying antirheumatic drugs (DMARDs), injectable biological DMARDs, targeted synthetic DMARDs, biologics, and small molecule drugs [9,10]. While these therapies can control disease activity and induce remission through frequent monitoring and treatment escalation, long-term use of immunosuppressants can cause significant side effects, and some patients remain unresponsive to existing treatments [[11], [12], [13]]. Therefore, there is a need for novel therapeutic approaches. Recent studies have highlighted mitochondrial dysfunction as a potential contributor to RA, through mechanisms such as increased ROS production, immune cells activation, and autoantibody production [[14], [15], [16], [17]]. Mitochondria thus emerge as a promising new therapeutic target for RA, although research in this area is still limited [18,19].

In this study, we integrate bioactive mitochondria with a cationic peptide dendrimer for targeted immunotherapy of RA. Mitochondrial transplantation has shown significant potential in treating various diseases such as nervous system and muscular system [[20], [21], [22]]. Unlike traditional treatments that primarily focus on symptom relief and disease management, mitochondrial transplantation aims to fundamentally repair cell function [[23], [24], [25]]. However, its application is limited by the invasive nature of the transplantation process and the potential loss of mitochondrial activity [26,27]. To address these challenges, targeted mitochondrial delivery may offer an effective solution. Dendritic polylysine, a positively charged cationic polymer, has garnered extensive attention as a drug transport carrier in recent years [28,29]. Its abundant primary amine groups can attract negatively charged inflammatory factors and cell debris, enhancing its therapeutic potential. Additionally, lysine, an essential amino acid, ensures good biocompatibility within the human body. Compared to traditional drug delivery methods, the cationic nature of dendritic polylysine enables it to target inflammatory sites, thereby improving drug bioavailability and reducing systemic toxicity [30].Despite these advantages, few studies have explored the efficacy of targeted mitochondrial delivery in RA therapy. It is conceivable to enhance mitochondrial transplantation by leveraging the unique properties of bioactive mitochondria and cationic peptide dendrimers.

**Fig. 1:**
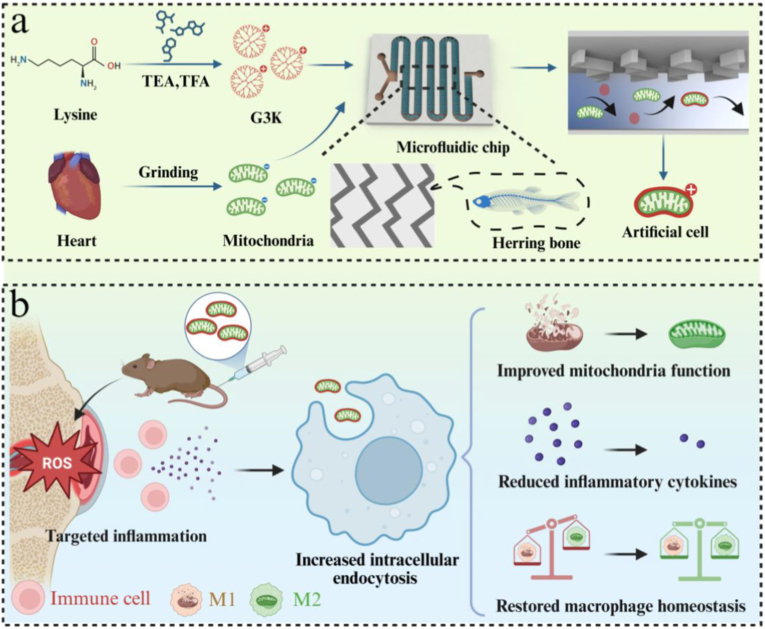
Schematic of Mito@G3K construction and the application in their targeted therapy for RA.

Herein, we developed core-shell biomimetic mitochondrial nanocomposite (Mito@G3K) by coating bioactive mitochondria with a third-generation polylysine dendrimer (G3K) nanogel, creating a biocompatible and stable mitochondrial transplantation system for targeted RA therapy. A microfluidic chip featuring curved S-shaped channels and a herringbone structure was used to generate a chaotic flow, enabling efficient production of the core-shell biomimetic mitochondrial nanocomposite (Mito@G3K) via electrostatic adsorption [31]. The Mito@G3K, with positively charged dendritic polylysine evenly distributed around bioactive mitochondria, significantly enhanced macrophage endocytosis, restored mitochondrial function, reduced M1 macrophage polarization, and lowered inflammatory factor levels. Importantly, these Miyo@G3K targeted joint sites in mice, effectively alleviating common RA symptoms, as evidenced by Micro-CT, joint histological, and ELISA analysis. Notably, mouse synovial transcriptome sequencing revealed significant improvements in mitochondrial metabolism-related pathways and effective inhibition of inflammatory pathways in the Mito@G3K group. Together, our findings suggest that microfluidic chip-based Mito@G3K offer a promising new approach for treating RA and other autoimmune diseases (see Fig. 1).

## 2 Results & discussion

**Fig. 2:**
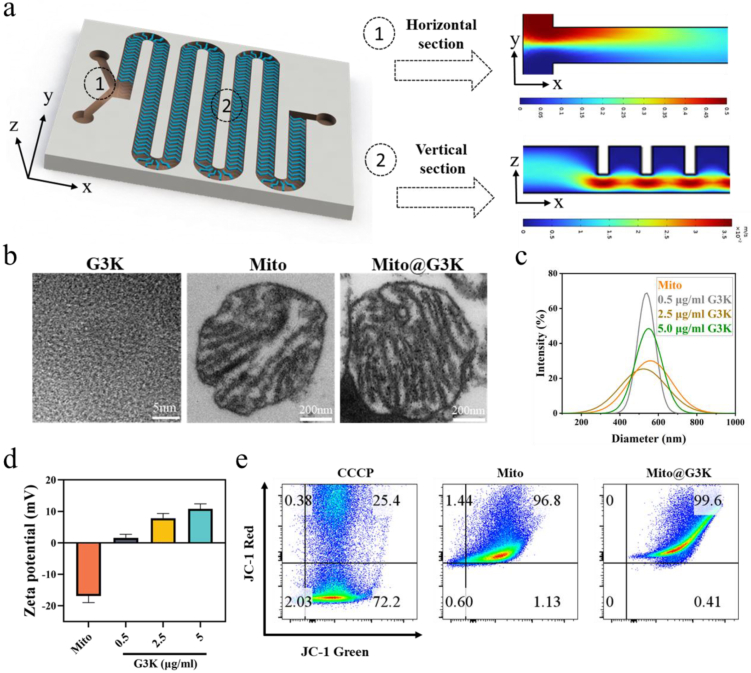
**Construction and characterization of Mito@G3K.** (a) Simulation analysis of fluid dynamics within the microfluidic chip. (b) Transmission electron microscope image of Mito@G3K. (c) Particle size distribution analysis of Mito@G3K. (d) Zeta potential analysis of Mito@G3K. (e) *Flow cytometry* analysis of mitochondrial membrane potential.

The third-generation of dendritic polylysine (G3K) was synthesized via solid-phase methods [[32], [33], [34]]. Specifically, G3K was synthesized from diphenylamine and BOC-protected lysine, catalyzed by triethylamine (TEA). Boc-protected lysine was added, and trifluoroacetic acid (TFA) was used for deprotection, removing all Boc groups to yield the third-generation dendritic polylysine. The successful synthesis of G3K was confirmed by ^1^H NMR (Fig. S1). Next, we isolated mitochondria from mouse myocardial tissue and confirmed their identity via Western blotting. The results showed that the mitochondrial markers TOM20 and VDAC1 were effectively separated from cytoplasmic markers (Fig. S2a). Endotoxin testing revealed that the level of endotoxins in the isolated mitochondria was very low, significantly below 0.03 EU/mL (Fig. S2b). To fabricate Mito@G3K, a microfluidic chip was designed and fabricated with curved S-shaped channels and a herringbone structure (Fig. S3). The S-channel facilitates extended mixing, while the herringbone structure generates turbulent fluid flow, enhancing contact between target biomolecules and improving reaction efficiency (Movie S1). Numerical simulation of the fluid streamline in the herringbone microchannel revealed rotational and distorted flow, as shown in the velocity streamline diagrams of the vertical and horizontal cross-section of the microfluidic channel (Fig. 2a). These results indicated that the herringbone channel generated chaotic advection, enhancing mixing efficiency. The morphology of the Mito@G3K was analyzed using transmission electron microscopy (TEM). The results showed that mitochondria retained their double-layer membrane structure, appearing elliptical or rod-like, with no significant morphological differences between the Mito@G3K and unmodified mitochondria (Fig. 2b). The particle size and zeta potential of the Mito@G3K were measured. Particle size analysis showed no significant changes in size (Fig. 2c), while zeta potential measurements indicated a shift from negative to positive potential with increasing dendritic polylysine content (Fig. 2d). To assess the activity of the extracted mitochondria, mitochondrial membrane potential was detected. *Flow cytometry* results showed that CCCP-induced apoptosis significantly decreased the membrane potential of normal mitochondria, whereas the membrane potential of the constructed Mito@G3K remained unchanged compared to that of normal extracted mitochondria (Fig. 2e). This indicates that the Mito@G3K can maintain good activity.

Supplementary data related to this article can be found online at https://doi.org/10.1016/j.mtbio.2026.103371

The following are the Supplementary data related to this article:

Multimedia component 1

**Fig. 3:**
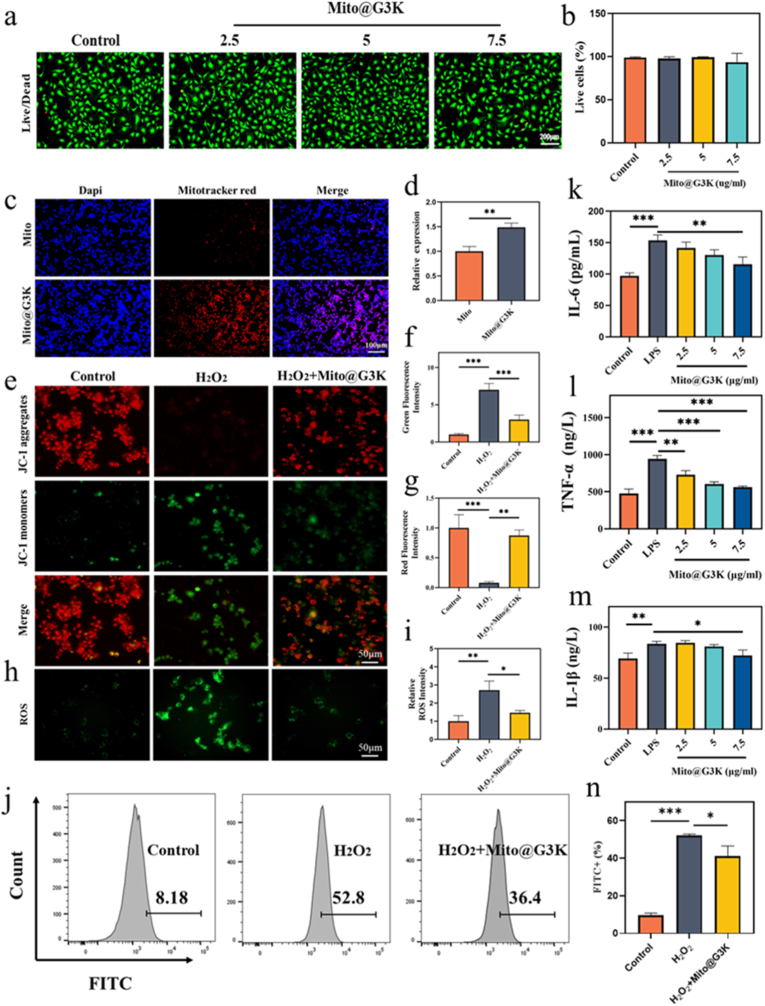
**Detection of the anti-inflammatory properties of Mito@G3K in vitro.** (a) Representative images of fluorescence staining of dead (red) and live (green) cells after co-culture with human umbilical vein endothelial cells. (b) *Statistical analysis* of cell survival and death (n = 3). (c) Immunofluorescence microscopy images showing mitochondrial uptake by macrophages. (d) Quantitative analysis of mitochondrial uptake in macrophages (n = 3). (e) Detection of mitochondrial membrane potential in macrophages stimulated with hydrogen peroxide. (f-g) *Statistical analysis* of mitochondrial monomers (f), mitochondrial aggregates (g) (n = 3). (h) Immunofluorescence imaging of ROS levels in macrophages stimulated by hydrogen peroxide. (i) *Statistical analysis* of cellular ROS fluorescence (n = 3). (j) *Flow cytometry* is used to analyze the ROS content of cells. (k-m) Mito@G3K reduced the secretion of inflammatory cytokines IL-6 (k), TNF-α (l) and IL-1β (m) in macrophages stimulated with LPS (n = 3). (n) *Statistical analysis* of the ROS content of cells determined by flow cytometry (n = 3). Data are expressed as mean ± SD. *P < 0.05, **P < 0.01, ***P < 0.001, using one-way ANOVA followed by a post hoc test. (For interpretation of the references to colour in this figure legend, the reader is referred to the Web version of this article.)

Then, we evaluated the biocompatibility of the Mito@G3K in vitro. Dead/live staining revealed that, compared to the blank control group, most cells maintained good viability after co-culture with the Mito@G3K, displaying their excellent biocompatibility (Fig. 3a–b). The efficient cellular internalization of mitochondria has long been a challenging issue. We successfully labeled the Mito@G3K with a mitochondrial membrane dye and observed their in vitro uptake by macrophages using immunofluorescence assay. The results showed that, compared to pure mitochondria, Mito@G3K were significantly more phagocytosed by macrophages within just 1 h, suggesting that the constructed Mito@G3K could enhance cell endocytosis (Fig. 3c–d). Furthermore, we marked the Mito@G3K sample with Mito tracker red and used Pico Green to label the mtDNA. Confocal microscopy results showed that both of them successfully integrated with the mitochondria of the host cells marked with Mito tracker deep red (Fig. S4a). To assess whether the constructed Mito@G3K could enhance mitochondrial metabolism, we stimulated macrophages with LPS. Following 24 h of LPS stimulation, ATP levels in macrophages decreased significantly. Notably, subsequent treatment with Mito@G3K led to a partial recovery of ATP levels (Fig. S4b). The Mito@G3K also showed strong anti-inflammatory and ROS resistance in vitro. Immunofluorescence results showed that the mitochondrial membrane potential of IBMDM macrophages stimulated by hydrogen peroxide was significantly reduced compared to the untreated group, but was notably restored after Mito@G3K transplantation (Fig. 3e–g). Furthermore, immunofluorescence analysis revealed that the level of reactive oxygen species (ROS) in IBMDM cells significantly increased after being stimulated by hydrogen peroxide, while it significantly decreased after Mito@G3K transplantation. (Fig. 3h–i). Similarly, flow cytometry showed that after being stimulated with hydrogen peroxide, the ROS levels in macrophages increased. After adding Mito@G3K, the ROS levels significantly decreased (Fig. 3j and n). Inflammatory cytokines such as TNF-α, IL-6 and IL-1β play crucial roles in inflammation. PCR results showed that the expression of TNF-α, IL-6 and other inflammatory factors was significantly upregulated after LPS stimulation in vitro, but was significantly downregulated after the addition of Mito@G3K, showing a dose-dependent trend (Fig. S5a–b). CD86, a signature marker of M1 macrophages, was significantly upregulated after LPS stimulation but was notably downregulated after the addition of Mito@G3K, suggesting that Mito@G3K can inhibit M1 macrophage differentiation in vitro (Fig. S5c). Meanwhile, flow cytometry results showed that LPS stimulation induced macrophage polarization toward the M1 macrophage, accompanied by an increase in the M1/M2 ratio. Following the addition of Mito@G3K, the M1/M2 ratio decreased (Fig. S6a–b). Immunofluorescence analysis yielded consistent results, demonstrating that Mito@G3K reduced the proportion of CD86-positive M1 macrophages (Fig. S6c–d) while increasing the proportion of CD206-positive M2 macrophages (Fig. S6e–f). ELISA analysis also showed that the expression of TNF-α, IL-1β, IL-6 and other inflammatory factors was significantly reduced after Mito@G3K treatment, consistent with the PCR findings (Fig. 3k–m).

**Fig. 4:**
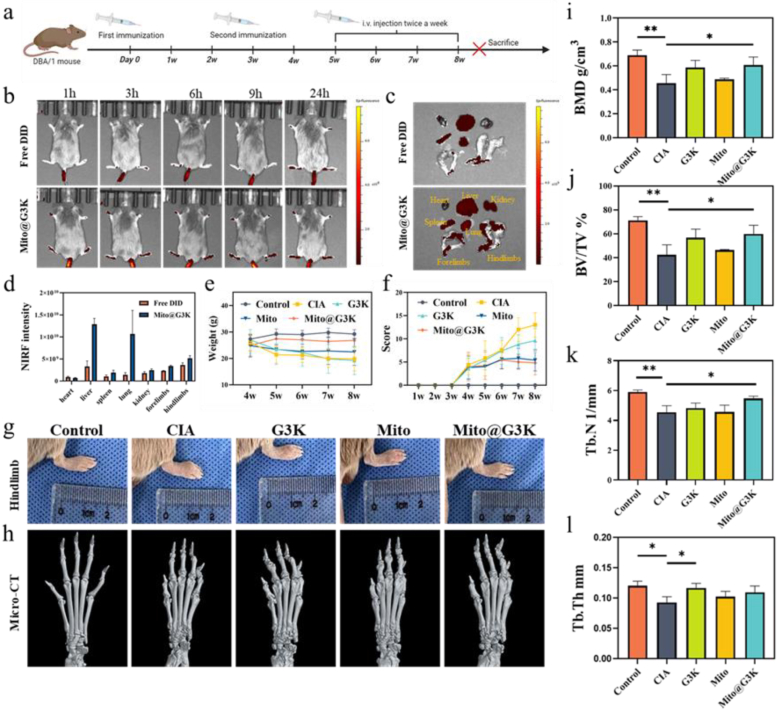
**Mito@G3K target the joints of CIA mice.** (a) Schematic diagram of Mito@G3K-based mitochondrial transplantation. (b) In vivo distribution of Mito@G3K in mice at different time points post-transplantation, visualized by small animal live imaging. (c) NIRF imaging of Mito@G3K distribution in major organs of mice. (d) Quantitative analysis of NIRF intensity in major organs of mice (n = 3). (e) The changes in the body weight of each group of mice (n = 5). (f) The scores for joint swelling of each group of mice (n = 5). (g) Representative bright-field images of joints from each group of mice. (h) Representative Micro-CT images of joints from each group of mice. (i-l) Quantitative analysis of joint bone mineral density (BMD) (i), bone volume fraction (BV/TV) (j), trabecular number (Tb.N) (k) and Trabecular thickness (Tb. Th) (l) in each group of mice (n = 3). Data are expressed as mean ± SD. *P < 0.05, **P < 0.01, ***P < 0.001, using one-way ANOVA followed by a post hoc test.

To further explore the therapeutic effects of Mito@G3K in vivo, we constructed a collagen-induced arthritis (CIA) mouse model. Mice received their first immunization on day 7 and a booster immunization on day 21 (Fig. 4a). By day 28, joint swelling was observed, indicating successful modeling. To test the targeting ability of Mito@G3K to inflammatory sites, mitochondria was labeled with DID membrane dye and injected into CIA mice, using free DID dye as a control. In vivo imaging revealed that Mito@G3K aggregated faster and more significantly at the limb joints of mice compared to free DID dye (Fig. 4b). Tissue and organ imaging showed that most mitochondria were taken up by the liver, and Mito@G3K were concentrated in the limbs (Fig. 4c). Quantitative fluorescence intensity analysis showed significantly higher fluorescence in the limbs and joints of mice treated with Mito@G3K compared to the free dye group (Fig. 4d), suggesting targeted delivery to inflammatory sites. Furthermore, we conducted rapid cryostat section staining of the joints of the mice. The results showed that there was a significant enrichment of mitochondrial signals in the joints of the mice, and these signals could be engulfed by macrophages (Fig. S7). To assess therapeutic efficacy, mice were divided into five groups including healthy control, CIA, G3K, Mito, and Mito@G3K therapy. Treatments were administered twice a week for 4 weeks, with PBS used for CIA group. Results showed that CIA mice experienced significant weight loss compared to healthy controls, while treatment groups regained weight (Fig. 4e). In addition, the clinical disease score of CIA mice increased over time, whereas scores in treatment groups rose slowly and eventually declined, with Mito@G3K group showing the most promising outcomes (Fig. 4f). In addition to observing significant paw swelling in CIA mice, we noted that paw swelling was significantly reduced in Mito@G3K therapy group, approaching levels seen in healthy, unmodeled mice (Fig. 4g). To further evaluate disease severity, we evaluated bone damage in the paws using micro-CT (Fig. 4h). Results showed severe joint damage in CIA mice, with significant reductions in bone mineral density (BMD), bone volume fraction (BV/TV), trabecular number (Tb.N) and trabecular thickness (Tb.Th) compared to healthy mice. Treatment groups showed some recovery, with Mito@G3K group demonstrating the best therapeutic effect (Fig. 4i–l).

**Fig. 5:**
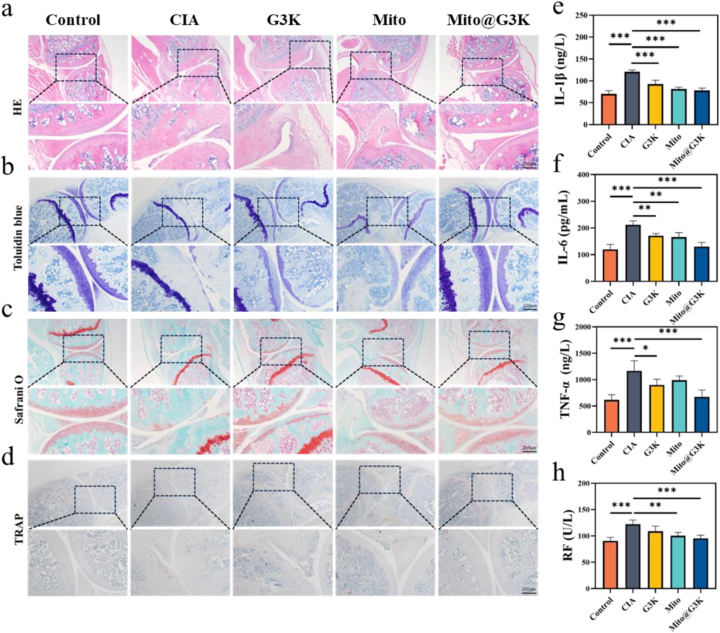
**Mito@G3K ameliorates bone and cartilage damage in CIA mice.** (a) Representative H&E staining images of joints from each group. (b) Representative toluidine blue staining images of joints from each group. (c) Representative Saffron solid green staining images of joints from each group. (d) Representative Tartrate-Resistant Acid Phosphatase staining images of joints from each group. (e-h) ELISA was used to determine the concentrations of IL-6 (e), IL-1β (f), TNF-α (g) and RF (h) in the mouse plasma (n = 5). Data are expressed as mean ± SD. *P < 0.05, **P < 0.01, ***P < 0.001, using one-way ANOVA followed by a post hoc test. (For interpretation of the references to colour in this figure legend, the reader is referred to the Web version of this article.)

In order to obtain a more intuitive assessment of the joint damage in mice, we conducted tissue section staining on the mice's joints. Histological analysis via HE staining revealed significant synovial hyperplasia and inflammatory cells infiltration in the CIA group, leading to bone erosion and destruction. In contrast, joint inflammation was controlled in the treatment groups, particularly in Mito@G3K group, which showed near-normal joint histology (Fig. 5a). Saffron solid green and Toluidine blue staining showed severe cartilage damage in CIA mice, which was significantly reduced in Mito@G3K group (Fig. 5b–c). Tartrate-Resistant Acid Phosphatase (TRAP) staining showed abnormal osteoclast activation in CIA mice, which was diminished in Mito@G3K group (Fig. 5d). Similar therapeutic effects were observed in ankle joint tissue sections (Fig. S8). Notably, ELISA analysis of mouse plasma indicated elevated levels of IL-6, IL-1β, TNF-α, and RF in CIA mice, which were significantly reduced in the treatment group (Fig. 5e–h).

**Fig. 6:**
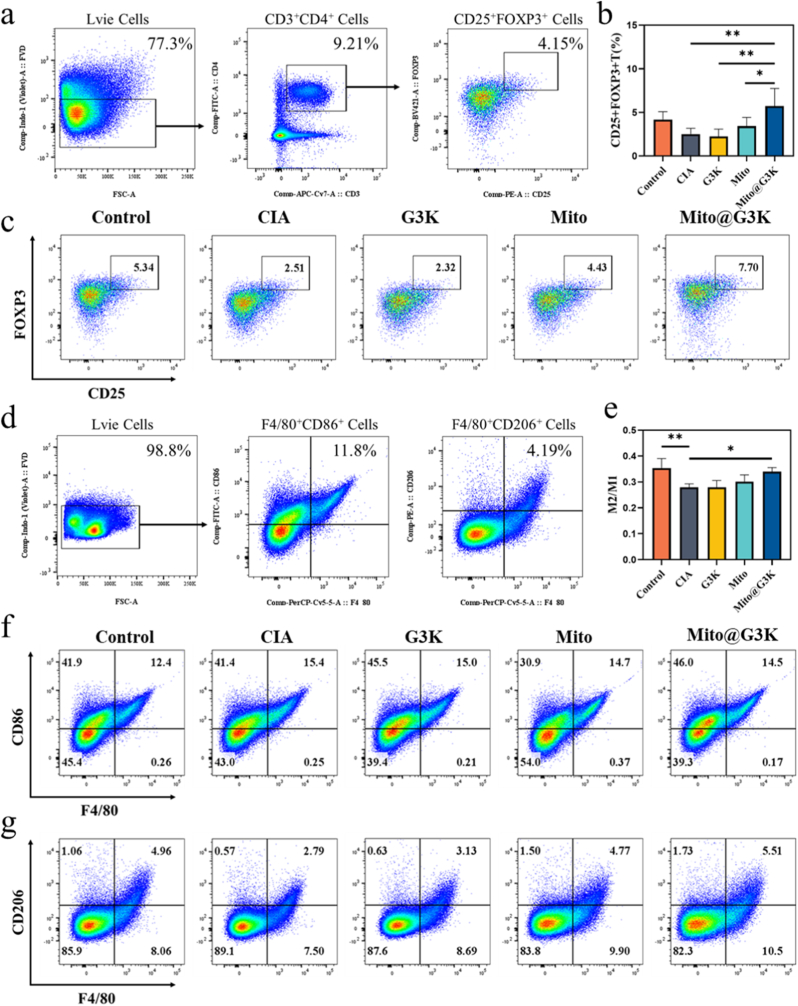
**Assessment of inflammation levels in different groups of mice.** (a) *Flow cytometry* gating diagram of TREG cells in mouse spleen cells. (b) Quantitative analysis of Treg cells in spleen cells from each group (n = 5). (c) Proportion of Treg cells in spleen cells from each group of mice. (d) *Flow cytometry* gating diagram of macrophages in mouse spleen cells. (e) Ratio of M2 to M1 macrophages in spleen cells from each group (n = 5). (f-g) Proportion of M1 (f) and M2 (g) macrophages in spleen cells from each group of mice. Data are expressed as mean ± SD. *P < 0.05, **P < 0.01, ***P < 0.001, using one-way ANOVA followed by a post hoc test.

Immune cell imbalance is crucial in RA development. Regulatory T cells (Tregs) are a subgroup of T cells that maintain immune tolerance and prevent autoimmune responses. Reduced Treg expression is reported in RA patients. Our results suggest that the increased proportion of Treg cells in Mito@G3K group may contribute to immunomodulation (Fig. 6a–c). Macrophages, key players in the innate immune system, are categorized into pro-inflammatory M1 and anti-inflammatory M2 types. *Flow cytometry* analysis of spleen macrophages showed that, compared to healthy mice, CIA mice had reduced M2 macrophage proportions and M2/M1 ratios, with no significant change in M1 (Fig. S9). Treatment groups, especially Mito@G3K group, exhibited increased M2 macrophage proportions and M2/M1 ratios, nearing normal levels (Fig. 6d–g).

**Fig. 7:**
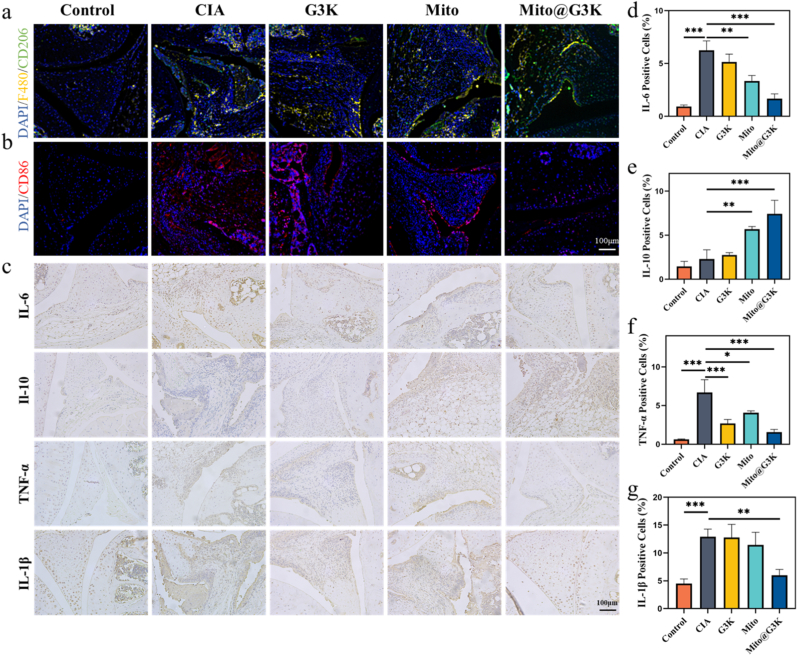
**Immunohistochemical analysis of mouse knee joint.** (a) Immunofluorescence staining image of M2 macrophages in the synovial membrane of mouse joints. (b) Immunofluorescence staining image of M1 macrophages in the synovial membrane of mouse joints. (c) *Immunohistochemical staining* of inflammatory cytokines in the knee joints of mice from each group. (d-g) Quantitative analysis of immunohistochemical staining for IL-6 (b), IL-10 (c), TNF-α (d), and IL-1β (e) in the knee joints of mice from each group (n = 3). Data are expressed as mean ± SD. *P < 0.05, **P < 0.01, ***P < 0.001, using one-way ANOVA followed by a post hoc test.

Subsequently, we also conducted an analysis of the infiltration of macrophages in the synovial tissue of the mice's joints. Immunofluorescence analysis showed that the proportion of M2 macrophages in the synovial membrane of the mice's joints increased (Fig. 7a and Fig. S10a), while the proportion of M1 macrophages decreased (Fig. 7b and Fig. S10b). Then, immunohistochemical staining was employed to analyze the expression of inflammatory factors in the joints of mice across different groups (Fig. 7c). The results showed that IL-6, TNF-α, IL-1β were highly expressed in the joints of CIA mice, whereas their expression was decreased in the treatment groups (Fig. 7d and 7f–g). IL-10, a potent anti-inflammatory cytokine that suppresses excessive inflammatory responses by inhibiting the production of pro-inflammatory cytokines and chemokines, was found to be significantly upregulated in the synovial tissue of mice treated with Mito@G3K, as shown by immunohistochemical staining (Fig. 7e).

**Fig. 8:**
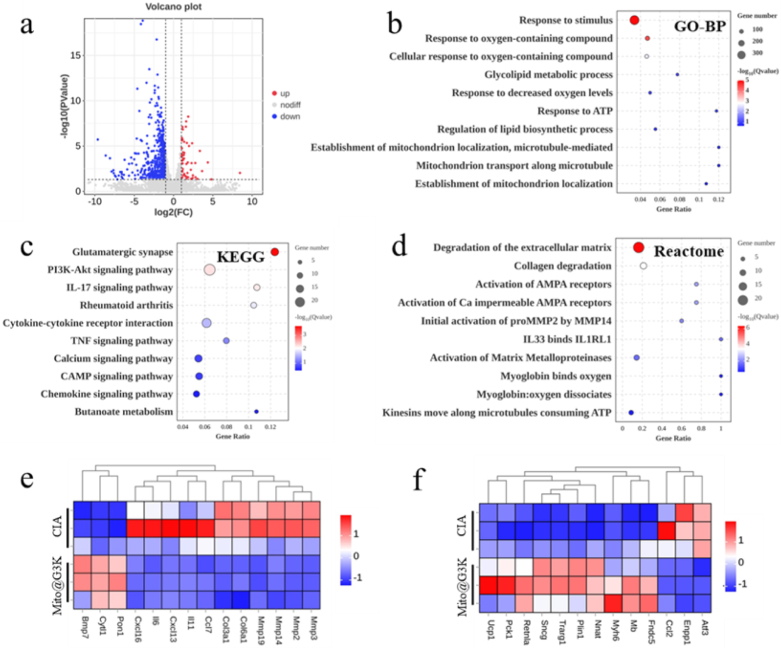
**Transcriptome sequencing analysis of synovial tissue from mouse joints.** (a) Differential gene expression volcano plot of synovial tissue from mice in Mito@G3K group compared to the CIA group. (b) Enrichment analysis of Gene Ontology - Biological Process (GO-BP) pathways for differentially expressed genes in synovial tissue from Mito@G3K group and CIA group. (c) Enrichment analysis of KEGG pathways in synovial tissue from mice in Mito@G3K group and CIA group. (d) Enrichment analysis of Reactome pathways for differentially expressed genes in synovial tissue from Mito@G3K group and CIA group. (e-f) Heatmaps of differentially expressed genes in synovial tissue from mice in Mito@G3K group and CIA group.

To further elucidate the therapeutic effects and underlying pathways of Mito@G3K, transcriptome sequencing was performed on the synovial tissue of mouse joints. The results showed that, compared to the disease group, there were 55 upregulated genes and 550 downregulated genes (*P* < 0.05, |log2 (fold change)| > 1) in Mito@G3K group (Fig. 8a). GO enrichment analysis revealed significant differences between the CIA group and Mito@G3K group in terms of “response to oxygen-containing compounds”, “glucolipid metabolism”, “response to low oxygen level” and “mitochondrial transfer through microtubules” (Fig. 8b and Fig. S11). KEGG signaling pathway enrichment showed significant differences in the PI3K-Akt signaling pathway, between CIA mice and those treated with Mito@G3K (Fig. 8c). We validated this signaling pathway using Western blotting. The results showed that the expression levels of P-PI3K and P-AKT in the synovial tissues of CIA mice were significantly higher than those in healthy mice. However, after treatment with Mito@G3K, these expression levels were reduced (Fig. S12). Reactome signaling pathway analysis suggested that the therapeutic effect of Mito@G3K may be related to the activation of AMPA receptor and matrix metalloproteinases (Fig. 8d). TRARG1 is a gene related to the transport regulation of GLUT4 (glucose transporter 4), mainly involved in the uptake and metabolic regulation of glucose by cells, thereby affecting mitochondrial energy production and oxidative stress levels. Heatmap results showed that TRARG1 expression was significantly increased in Mito@G3K therapy group. FNDC5, a gene encoding a secreted protein containing the fibronectin III domain, was significantly upregulated after Mito@G3K treatment, as shown by heatmap analysis. Depletion of FNDC5 results in decreased mitochondrial membrane potential (ΔΨm) and ATP content, as well as increased ROS levels.

Additionally, heatmap results showed that many genes associated with RA inflammation and bone destruction, such as IL-6, MMP3, and CXCL13, were significantly reduced after Mito@G3K treatment, while anti-inflammatory genes (e.g. Pon1, Cytl1) and osteogenic genes (e.g. Bmp7) were upregulated (Fig. 8e–f). In summary, Mito@G3K may regulate inflammatory responses in CIA mice by improving mitochondrial metabolism. To assess the biocompatibility of Mito@G3K, we measured blood routine and liver function in mice from each group, finding no significant abnormalities. No significant lesions were observed in major organs such as the heart, liver, spleen, lung, and kidney, suggesting low toxicity and good biocompatibility of Mito@G3K (Fig. S13a–b). To assess the potential immunogenicity of exogenous mitochondria, we measured the plasma levels of anti-mitochondrial antibodies (AMA) and anti-double-stranded DNA (dsDNA) antibodies in each group of mice using ELISA (Fig. S13c).

## 3 Conclusion

In this work, we successfully developed the Mito@G3K based on a microfluidic chip. The microfluidic chip features a curved S-shaped channel topped with a herringbone structure, which induces chaotic flow and ensures thorough mixing of the liquid. Our data demonstrated the anti-inflammatory potential of Mito@G3K in vitro, revealing their ability to effectively inhibit LPS-induced macrophage inflammation. The engineered modification of dendritic polylysine enables mitochondria to target sites of inflammation, thereby facilitating precise mitochondrial delivery. Mito@G3K also exhibited promising therapeutic outcomes in vivo. Their natural amino acid composition ensures minimal toxicity within the body. In vivo imaging of small animals confirmed that Mito@G3K transplantation can target joint inflammation sites in mice. Histological staining, flow cytometry, ELISA, and transcriptome sequencing results collectively showed that Mito@G3K significantly ameliorated inflammation in CIA mice. These findings suggest that microfluidic chip-based Mito@G3K transplantation may represent a potential new therapeutic approach for RA.

Despite the evident advantages of this Mito@G3K-based delivery system, several areas warrant further improvement. First, in terms of Mito@G3K design, an intelligent shell design could extend the survival time of mitochondria in the body. Incorporating “don't eat me” and “homing” signals could reduce immune clearance and systemic side effects. Second, cationic dendritic polylysine requires further functional groups modifications to achieve more stable binding and modification. Third, given the complexity of the human metabolic cycle, a deeper understanding of biological interactions is needed to integrate these insights into our drug delivery systems.

In conclusion, while mitochondrial transplantation holds great promise as a potential therapeutic approach for future clinical application, several key challenges must be addressed before it can be successfully translated into practice. First and foremost, the safety and ethical issues associated with this technique require thorough evaluation. Second, a stable supply of donor mitochondria and standardized product preparation processes are essential. Furthermore, rigorous quality control measures for the final product are also necessary.

## 4 Experiment

*Materials:* Tissue mitochondrial separation kit, mitochondrial membrane potential detection kit, mito-tracker, picogreen and mitochondrial membrane dye were obtained from Beyotime Biotechnology. Bovine collagen Type II and full/partial Freund's adjuvants were purchased from ThermoFisher. Mouse ELISA test kits were purchased from Jiangsu Jingmei. Murine flow fluorescent antibodies were obtained from BD or Biosciences. Mouse IL-10, IL-1β, TNF-α, IL-6, TOM 20, VDAC1, PI3K, AKT and α-tubulin antibodies were purchased from Proteintech.

*Isolation of mitochondria:* The extraction of mitochondria is carried out using the mitochondrial extraction kit. First, take the fresh heart tissue of the mouse and use scissors to cut it into small tissue fragments. Then, the tissue was digested for 20 min using trypsin digestion solution. Then add the mitochondrial extraction reagent and perform a 20-s centrifugation at 600*g* to remove the remaining trypsin. Then add the mitochondrial extraction reagent and use a glass homogenizer tube to carefully grind the tissue into a suspension. Then, 1000 g of the sample was centrifuged to remove the sediment. The supernatant was then subjected to another 3500 g centrifugation for 10 min, and the resulting sediment was the mitochondria.

*Identification of mitochondria and detection of endotoxins*: Add RIPA and PMSF to the extracted mitochondrial, and perform ice-cold lysis for 30 min. Then, centrifuge at 12000g for 10 min to remove the precipitate. Add the loading buffer and heat it at 100°C for 10 min. Then, different-sized proteins were separated by SDS-PAGE gel electrophoresis. Then, the protein on the gel was transferred to the PVDF membrane through an electric field. Use 5% skimmed milk for sealing. Then, the primary antibody was incubated overnight, and then it was co-incubated with the corresponding secondary antibody at room temperature for 2 h. Finally, the imaging instrument (Bio-Rad) is used to develop the membrane. The detection of endotoxin was carried out using an endotoxin detection kit (laboratory mouse lysis gel method, with a sensitivity of 0.030 EU/ml, Beyotime). After adding the endotoxin detection reagent, observe the flow state of the gel to determine the level of endotoxin.

*Preparation of microfluidic chip and Mito@G3K:* AutoCAD was used to design a sketch of the chip and a corresponding mask was created. A silicon wafer was cleaned with a plasma cleaner for 60 s, followed by even coating with photoresist and heating at 90°C for 30 min. After cooling to room temperature, the chip pattern was printed onto the wafer. The residual photoresist was removed with ethyl lactate, and fry the film was baked at 110°C for 20 min. PDMS solution (10:1, w/w) was added to the mold, degassed using a vacuum pump, and left wo set overnight. The solidified PDMS blocks were then extracted, sterilized by overnight UV irradiation, and sealed by plasma treatment for 30-45 s. The PDMS surfaces were oxidized with a plasma cleaner to form O-Si-O covalent bonds, and the upper and lower PDMS were bonded together to obtain the microfluidic chips. Next, the G3K solution (2.5 mg/ml) and the mitochondrial solution (0.5 mg/ml) were introduced into the microfluidic chip inlet via a peristaltic pump at a flow rate of 1 ml/min. The resulting product was Mito@G3K.

*Reactive oxygen species (ROS) measurements:* iBMDM cells (5.0 × 10^5^ cells/mL) were seeded in 24-well plates and cultured overnight for cell adherence. Mito@G3K-pretreated (7.5 μg/ml) cells were added for 1 h and H_2_O_2_ (400 μm) was added for 4 h. The stimulated cells were incubated with DCFH-DA (10 μM) dye at 37°C for 20 min. After incubation, excess dye was removed with PBS, and fluorescence images were captured using a fluorescence microscope (Leica). Quantitative analysis was performed using ImageJ software. The method for detecting the intensity of ROS by flow cytometry is the same as described above. The cell suspension was subjected to flow cytometry analysis using the BD FACS Calibur (BD, New Jersey, USA). Data were analyzed using FlowJo software.

*Mitochondrial membrane potential (MMP) analysis:* Oxidative stress was induced in cells with 400 μM H_2_O_2_, followed by incubation with JC-1 fluorescent dye (10 μM) at 37°C for 20 min. The cells were then washed with cold PBS. Fluorescence images of JC-1 aggregates (red) and monomers (green) were captured using a Leica fluorescence microscope (Leica), and their intensities were measured using ImageJ software.

*Cell uptake assay:* Mito@G3K was labeled with fluorescent staining using mitochondrial tracker (Beyotime Biotechnology). RAW cells were cultured overnight to ensure full adherence. The fluorescent labeled Mito@G3K were co-cultured with RAW cells, and fluorescence microscopy (Leica) was used to capture images. The fusion of Mito@G3K with the host cell mitochondrial network was visualized using confocal fluorescence microscopy. First, Mito@G3K was co-incubated with MitoTracker Red (diluted 1:2000) and PicoGreen (diluted 1:200) at 37°C for 30 min to label the Mito@G3K particles. The labeled Mito@G3K was then washed with PBS. Meanwhile, RAW cells were co-incubated with MitoTracker Deep Red (diluted 1:1000) at 37°C for 30 min to stain their mitochondria, followed by three washes with PBS. The labeled Mito@G3K was then added to the RAW cells and incubated overnight. Subsequently, the cells were incubated with Hoechst (10 μg/ml) at 37°C for 10 min to stain the cell nuclei. Finally, imaging was performed using confocal fluorescence microscopy (Zeiss LSM).

ATP content detection: iBMDM cells were seeded in 24-well plates and cultured overnight for cell adherence. Mito@G3K-pretreated cells were added for 1 h and LPS (1 μg/ml) was added for 24 h. The cells were lysed using the ATP detection lysate (Beyotime Biotechnology), and then the chemical luminescence intensity of each group was detected using an enzyme detector (Bio-tek) after adding the ATP detection reagent.

*Live imaging of small animals:* DID dye was used to label the Mito@G3K, which were then injected into CIA mice. Fluorescence intensity in the joints was captured at different time points using a live animal imaging system (IVIS Spectrum).

*Animal model induction and treatment:* Male DBA/1 mouse (six weeks) were purchased from the laboratory animal room of Nantong University. The CIA mouse model was established by intradermal injections of bovine type II collagen emulsified with Freund's adjuvant (1:1 vol ratio). The successful establishment of the CIA model was demonstrated by the observation of joint swelling in mice 28 days after immunization. Subsequently, the mice were divided into five groups: healthy control, CIA model, G3K treatment, mitochondrial treatment, and Mito@G3K treatment. Each group received the corresponding therapeutic agent via tail vein injection twice a week, with a volume of 100 μL per injection. The dosage per administration was as follows: 50 mg/kg body weight for the G3K group, 10 mg/kg for the mitochondrial group, and Mito@G3K at a mitochondrial-equivalent dose of 10 mg/kg for the Mito@G3K group. All animal experiments were conducted in accordance with the guidelines of the Animal Protection and Use Committee of Nantong University. All experiments were approved by the Animal Ethics Committee of Nantong University (S20240927-002).

*Immunohistochemical staining:* Mouse joint synovial tissue was embedded in paraffin wax and sectioned. Antigen retrieval solution was used to fully expose tissue antigen epitopes after dewaxing.

*Quantitative real-time PCR (qRT-PCR) analysis:* iBMDM cells were seeded in 24-well plates and cultured overnight for cell adherence. Mito@G3K-pretreated (7.5 μg/ml) cells were added for 1 h and LPS (1 μg/ml) was added for 4 h. RNA was extracted from treated cells using an RNA extraction kit (YISHAN BIOTECH). This RNA was reverse-transcribed using the reverse transcription kit from Vazyme Biotech in a PCR instrument (Bio-Rad), and then converted into cDNA. The resulting cDNA was amplified using a qPCR instrument (QuantSudio 5).

*Flow cytometry of mouse spleen:* Fresh mouse spleen tissue was minced and filtered to remove excess tissue residue. Cell suspensions were obtained by centrifugation and treated with red blood cell lysate to remove erythrocytes. For splenic macrophages, cells were stained at room temperature for 30 min with multiple anti-mouse monoclonal antibodies (BD or Biosciences): Fixable Viability Dye-455UV; F4/80 percp-cy5.5; CD86 FITC; CD206 PE; CD25 PE; FOXP3 BV421. Cells were fixed with IC fixation solution, incubated with CD206 PE antibody at room temperature for 30 min after membrane permeabilization. Subsequently, the cell suspension was subjected to flow cytometry analysis using the BD FACS Calibur (BD, New Jersey, USA). Data were analyzed using FlowJo software.

*Elisa:* iBMDM cells were seeded in 24-well plates and cultured overnight for cell adherence. Mito@G3K-pretreated (7.5 μg/ml) cells were added for 1 h and LPS (1 μg/ml) was added for 24 h. Then, the cell supernatant was taken and the expression of inflammatory factors was detected using the ELSIA kit (Jingmei, Jiangsu, China). The content of inflammatory factors in mouse plasma was measured by the ELISA kit (Jingmei, Jiangsu, China). The experiment was completed according to the instructions. After adding the ELISA detection termination solution, read the values using the microplate reader (Thermo Fisher) at 450 nm.

*Statistical analysis:* Experimental data were analyzed using *one-way* analysis of variance (ANOVA) test via GraphPad software where appropriate.

## CRediT authorship contribution statement

**Nengjie Yang:** Data curation, Methodology, Software, Writing – original draft. **Chen Dong:** Methodology. **Rui Zhao:** Investigation, Methodology. **Mei Yang:** Software, Supervision. **Shiwen Ni:** Software. **Yi Jin:** Software, Supervision. **Qingshui Wen:** Project administration, Resources. **Cong Xu:** Methodology. **Zhifeng Gu:** Conceptualization. **Yujuan Zhu:** Conceptualization. **Chi Sun:** Conceptualization.

## Declaration of competing interest

The authors declare no competing financial interests.

## Data Availability

Data will be made available on request.
