# Supplementary material for: Microfluidic-based biomimetic mitochondrial nanocomposite for targeted immunotherapy of rheumatoid arthritis via mitochondrial transplantation
Source: Mater Today Bio. 2026 Jun 19;39:103371. doi: 10.1016/j.mtbio.2026.103371 (PMC13312487; doi:10.1016/j.mtbio.2026.103371)
Supplement: Multimedia component 2 [file mmc2.docx]

**Supplementary Information**

**Microfluidic-based biomimetic mitochondrial nanocomposite for targeted immunotherapy of rheumatoid arthritis via mitochondrial transplantation**

Nengjie Yang^1^, Chen Dong^1^, Rui Zhao^1^, Mei Yang^1^, Shiwen Ni^1^, Yi Jin^1^, Qingshui Wen^1^, Cong Xu^2^, Zhifeng Gu^1,*^, Yujuan Zhu^1,*^, and Chi Sun^3,*^

^1^ Department of Rheumatology, Research Center of Clinical Medicine, Research Center of Immunology, Affiliated Hospital of Nantong University, Medical School of Nantong University, Nantong 226001, China

^2^ Department of Biomedical Engineering, Columbia University, New York, NY, 10027, USA.

^3^ Department of Geriatrics, Research Center of Clinical Medicine, Research Center of Immunology, Affiliated Hospital of Nantong University, Medical School of Nantong University, Nantong 226001, China

Correspondence: guzf@ntu.edu.cn (Z. Gu), yujuanzhu@ntu.edu.cn (Y. Zhu) and tdfy_sc@126.com (C. Sun)

**
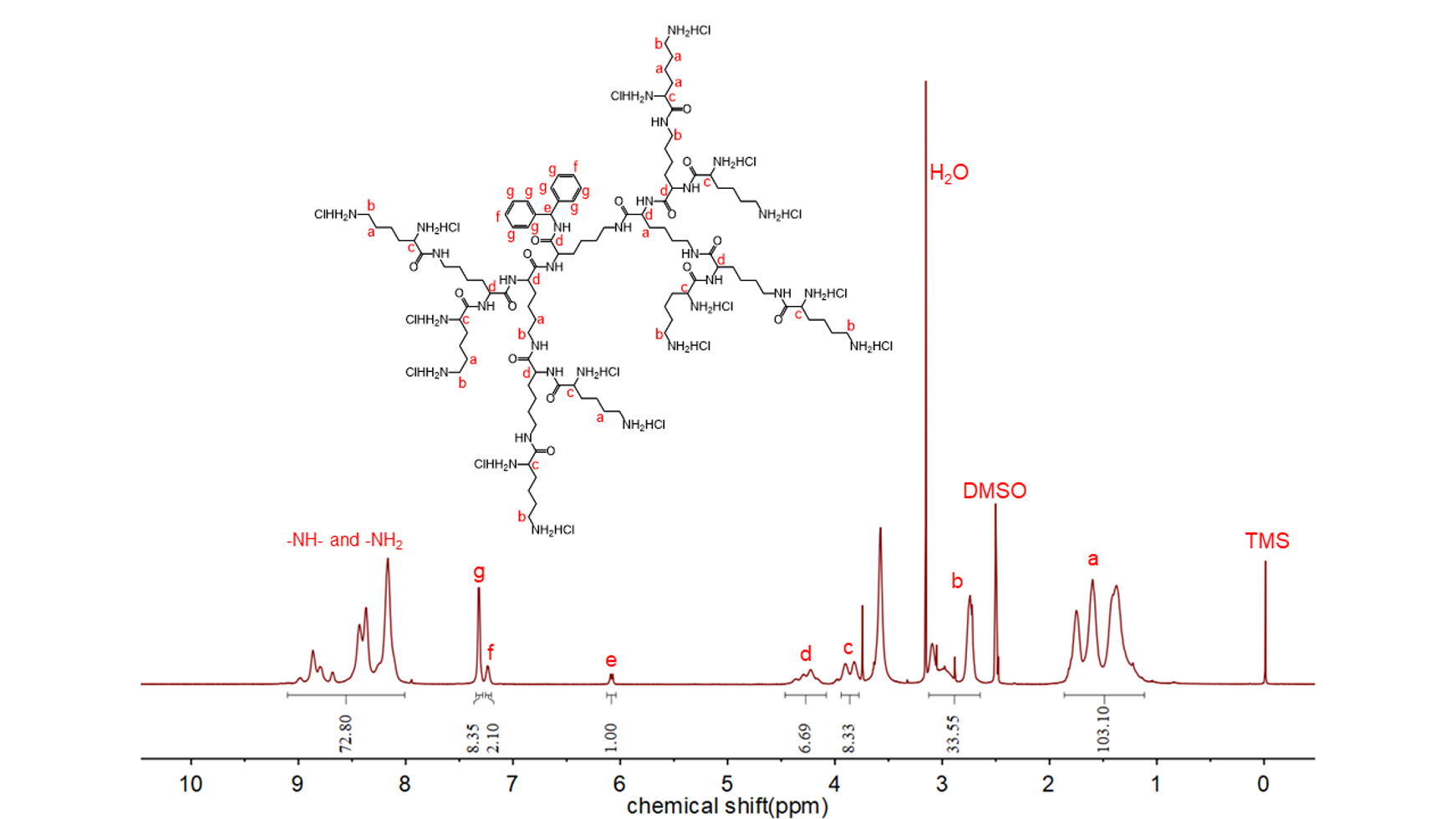
Figures**

**Figure S1. ^1^H-NMR spectra of G3K.**

**
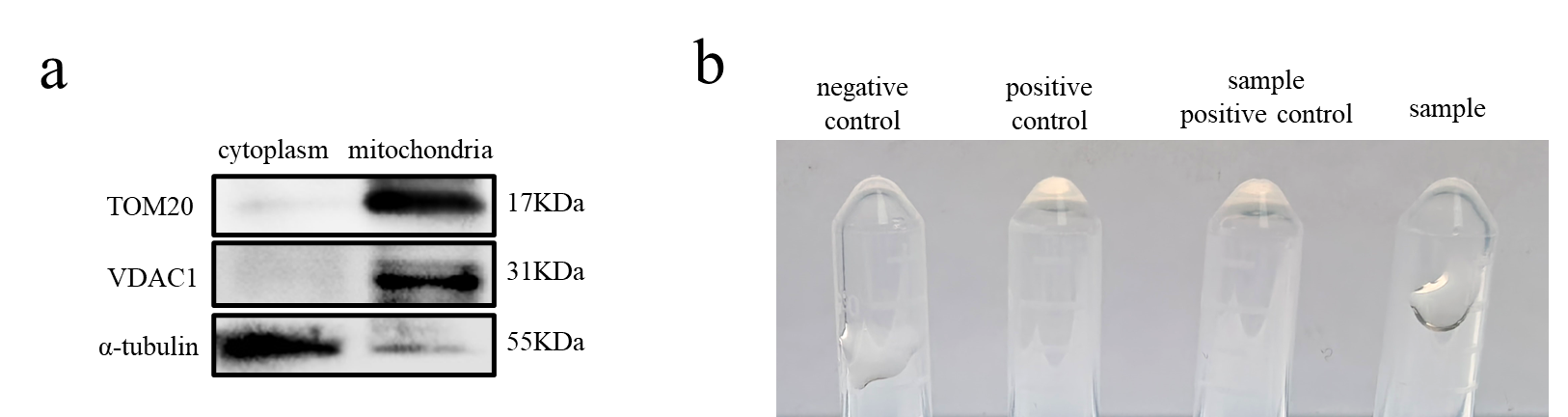
**

**Figure S2. Analysis of mitochondrial quality.** (a). Western blot identification of mitochondrial markers TOM20 and VDAC1. (b). Detection of endotoxin content in mitochondria.

**
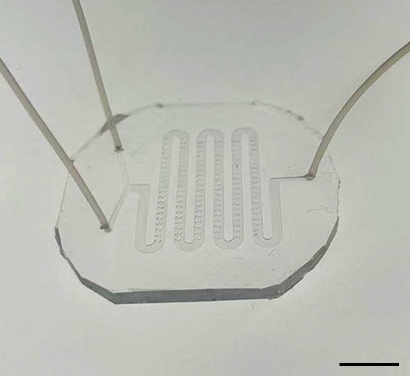
**

**Figure S3. Bright-field images of microfluidic chips.** **Scale bar, 1cm.**

**
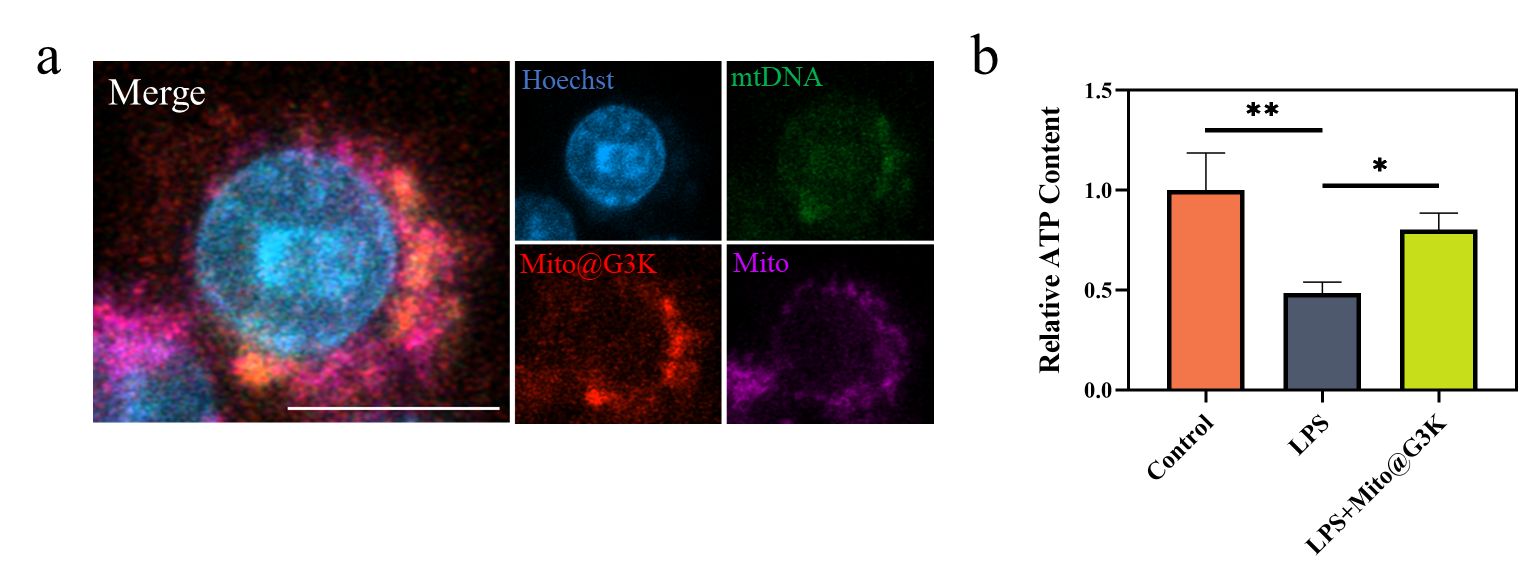
**

**Figure S4. Mito@G3K's fusion with the host cell.** (a). Confocal fluorescence images reveal the fusion of MitoTracker Red and Picogreen-labeled Mito@G3K into the host cell's mitochondrial network. Scale bar, 10 μm. (b). The ATP content within macrophages after the addition of LPS and Mito@G3K was detected using the ATP detection kit (n=3). Data are expressed as mean ± SD. *P < 0.05, **P < 0.01, ***P < 0.001, using one- way ANOVA followed by a post hoc test.

**
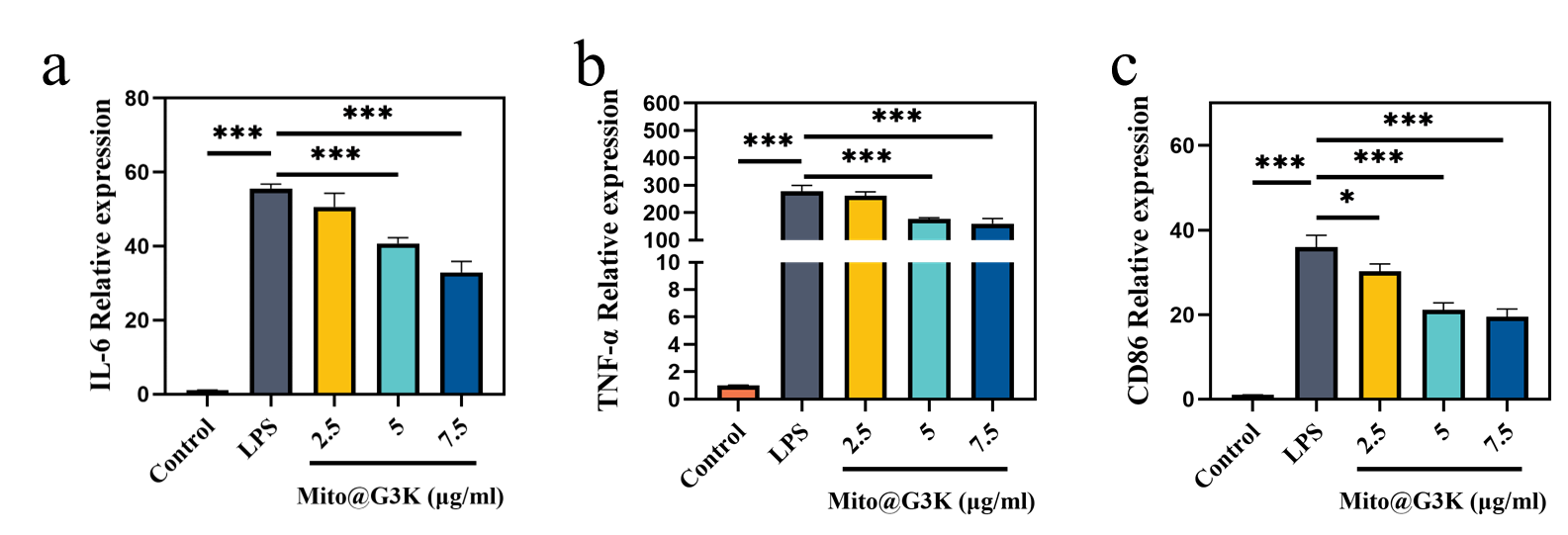
**

**Figure S5. The expression level of markers in M1 macrophages was detected by PCR.** Expression level of IL-6 (a), TNF-α (b) and CD86 (c) genes in macrophages (n=3). Data are expressed as mean ± SD. *P < 0.05, **P < 0.01, ***P < 0.001, using one- way ANOVA followed by a post hoc test.


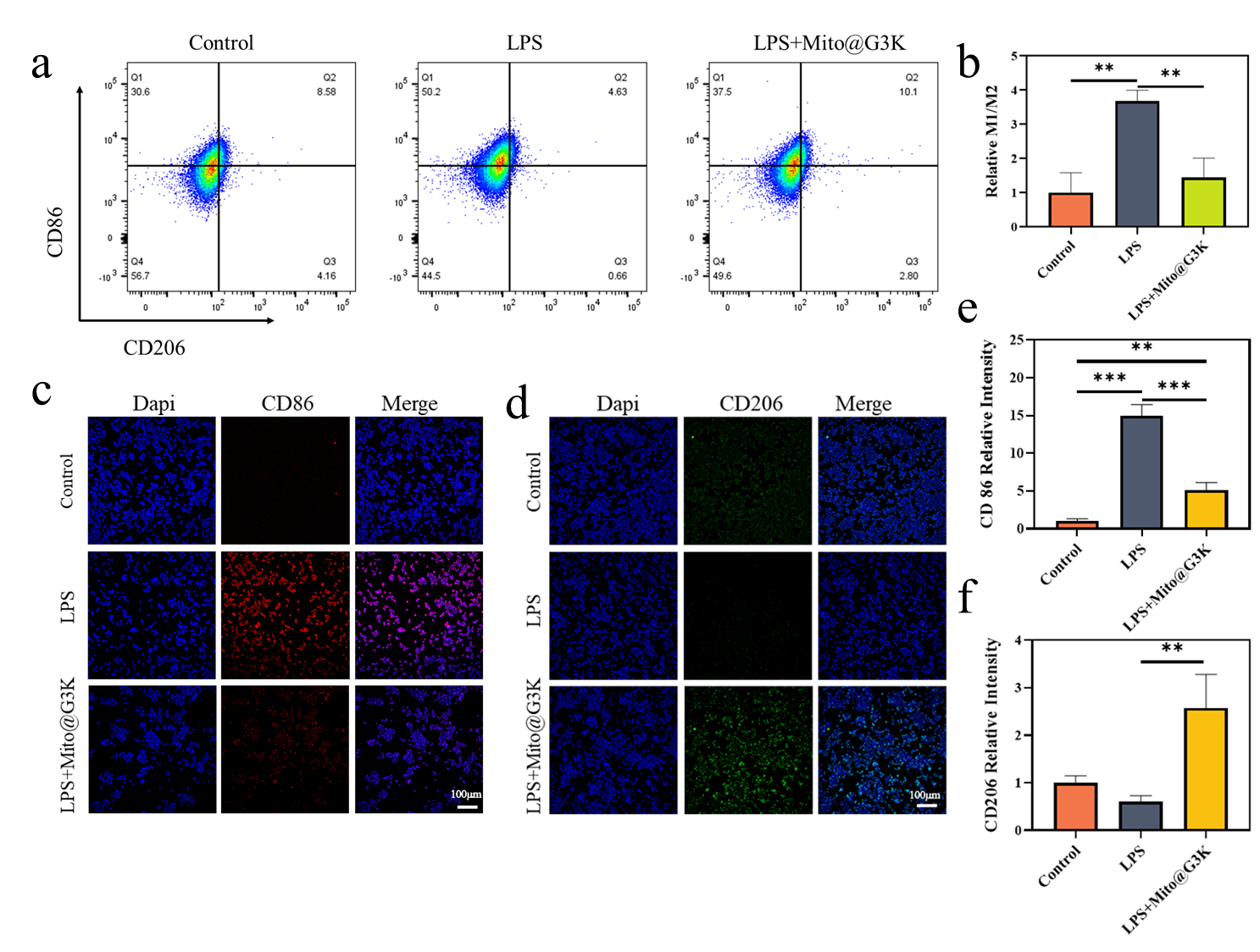
**Figure S6. Mito@G3K regulates macrophage polarization.** (a). Mito@G3K's influence on the polarization of macrophages and (b) the quantification of the ratio of M1/M2 macrophages(n=3). (c-d). Immunofluorescence evaluation of Mito@G3K and (e-f) quantitative assessment of macrophage polarization following LPS stimulation (n=3). Data are expressed as mean ± SD. *P < 0.05, **P < 0.01, ***P < 0.001, using one- way ANOVA followed by a post hoc test.


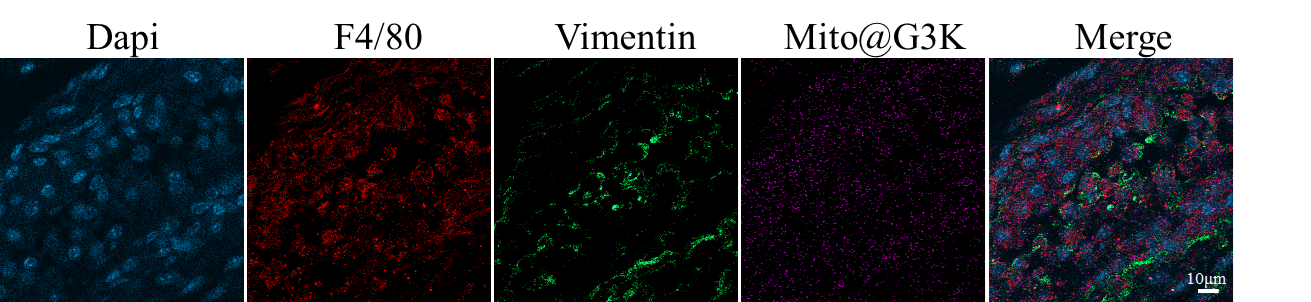


**Figure S7. The DIR-labeled Mito@G3K can reach the joints of mice and be phagocytosed by macrophages.**

**
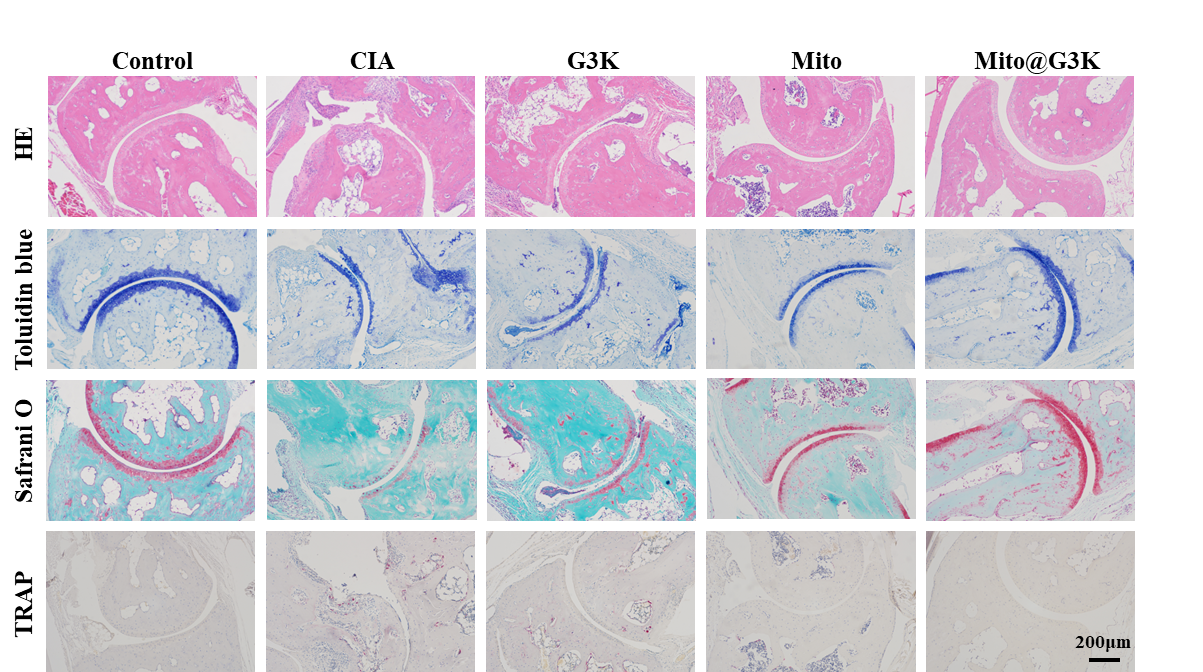
Figure S8. HE staining, toluidine blue, Safrani O and TRAP staining of the ankle joint of mice.**

**
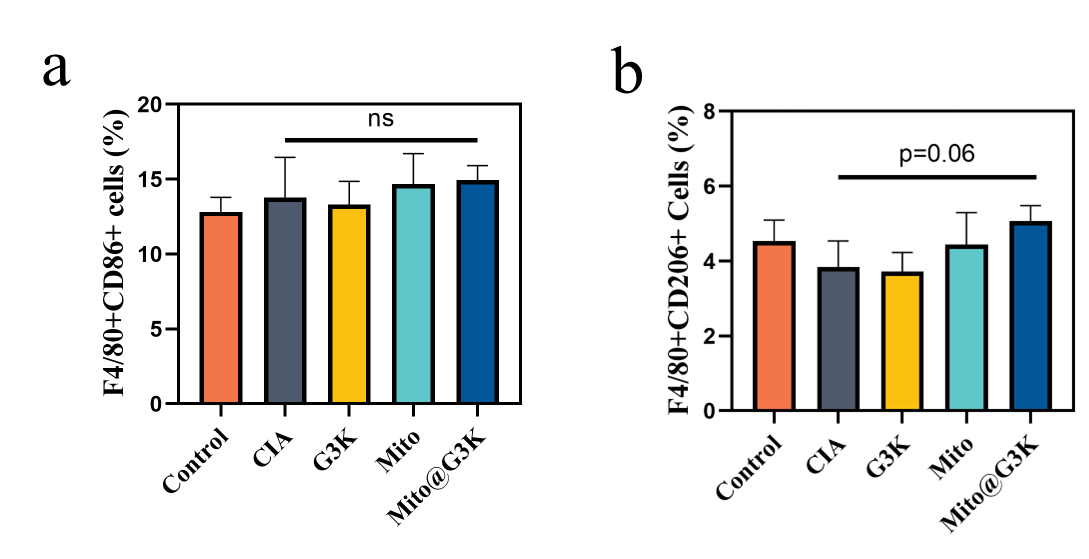
**

**F****igure S9. Statistical analysis of mouse splenic macrophages.** Flow cytometry analysis of M1 (a) and M2 (b) macrophages (n=5). Data are expressed as mean ± SD. *P < 0.05, **P < 0.01, ***P < 0.001, using one- way ANOVA followed by a post hoc test.


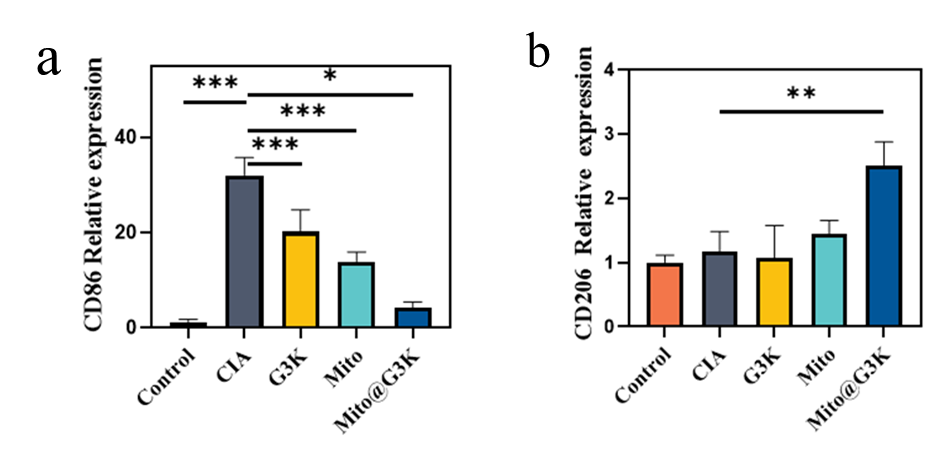


**Figure S10. Statistical analysis of mouse splenic macrophages.** Quantitative analysis of the immunofluorescence expression of CD86 (a) and CD206 (b) (n=3). Data are expressed as mean ± SD. *P < 0.05, **P < 0.01, ***P < 0.001, using one- way ANOVA followed by a post hoc test.

**
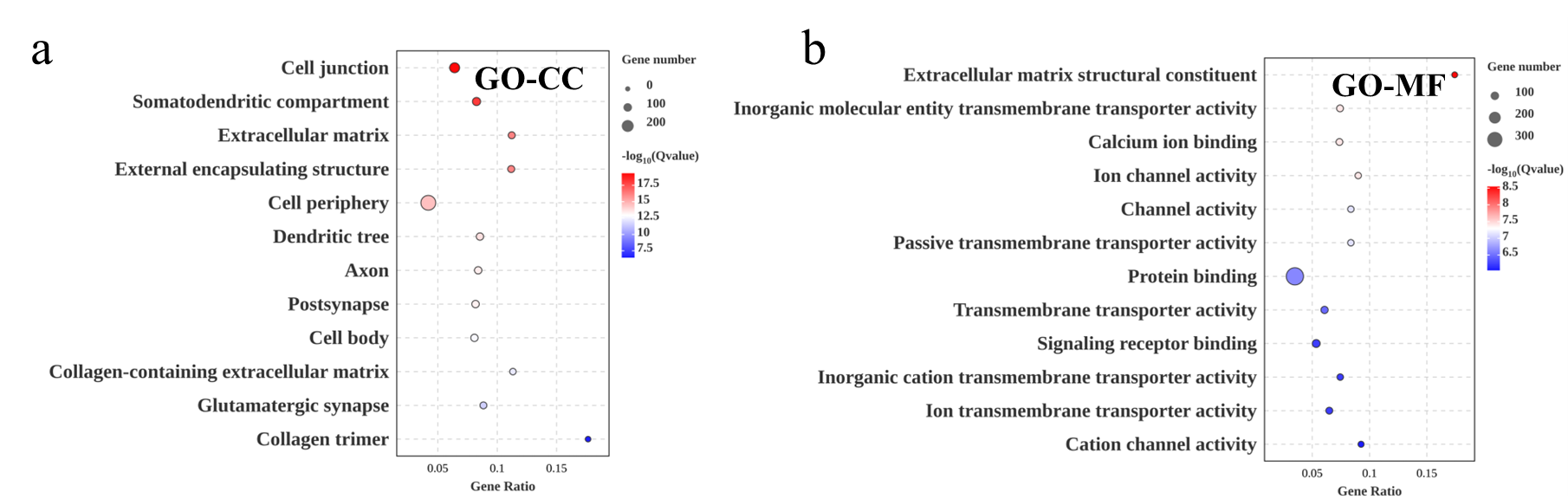
Figure S11. GO enrichment analysis of transcriptome sequencing in synovial tissue of mouse joints.**

**
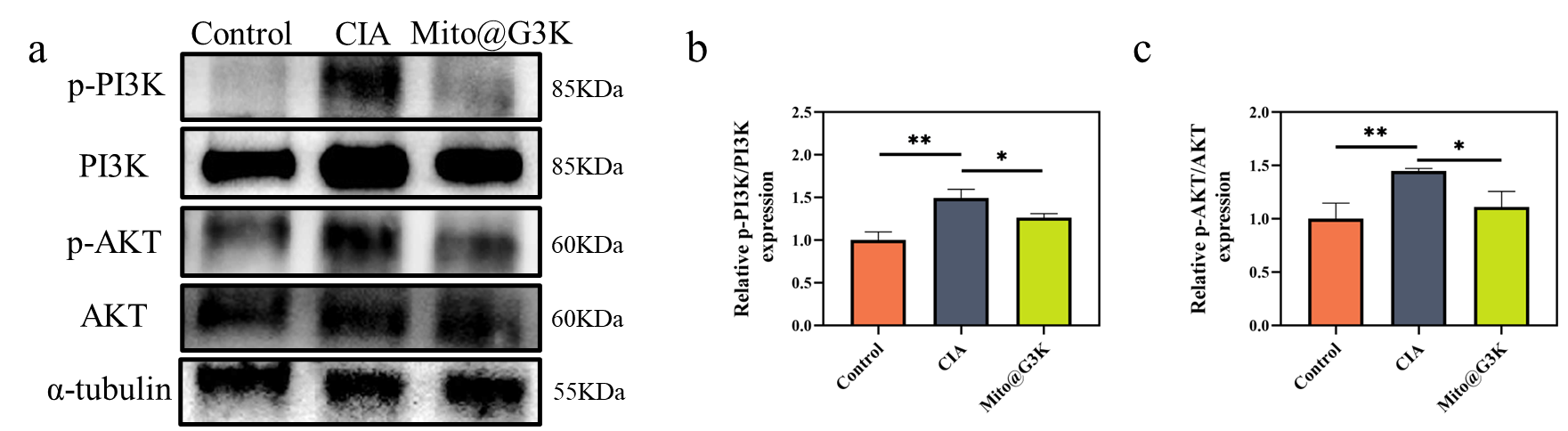
**

**Figure S12. Protein expression in the synovial membrane of mouse joints.** (a) The protein expressions of p-PI3K and p-AKT in the synovial membranes of mice were detected by WB, and the quantitative statistical analysis (b-c) of their contents was conducted (n=3). Data are expressed as mean ± SD. *P < 0.05, **P < 0.01, ***P < 0.001, using one- way ANOVA followed by a post hoc test.

**
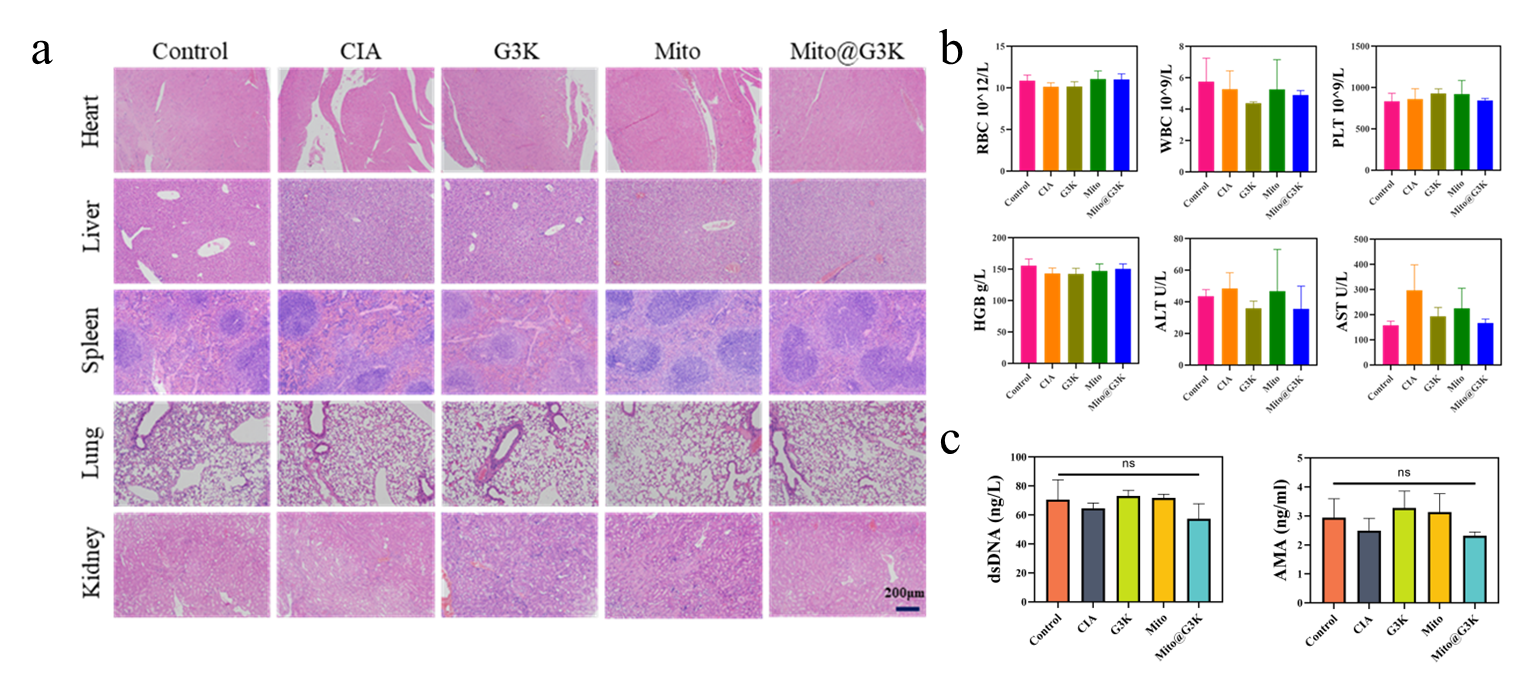
**

**Figure S13. Biocompatibility of artificial cells in mice.** (a) HE staining of major organs of mice in each group. (b) Blood routine and liver function in each group (n=3). (c) The detection results of dsDNA and AMA antibodies in the serum of each group of mice (n=5). Data are expressed as mean ± SD. *P < 0.05, **P < 0.01, ***P < 0.001, using one- way ANOVA followed by a post hoc test.
